# Renin-angiotensin system mechanism underlying the effect of auricular acupuncture on blood pressure in hypertensive patients with phlegm-dampness constitution: Study protocol for a randomized controlled trial

**DOI:** 10.1371/journal.pone.0294306

**Published:** 2024-02-01

**Authors:** Qianyin Zhu, Tingyu Mu, Die Dong, Lingshan Chen, Jiayi Xu, Cuizhen Shen

**Affiliations:** 1 School of Nursing, Zhejiang Chinese Medical University, Hangzhou, Zhejiang Province, China; 2 School of Nursing, Anhui Medical University, He Fei Shi, Anhui Province, China; Kwame Nkrumah University of Science and Technology Faculty of Pharmacy and Pharmaceutical Sciences, GHANA

## Abstract

**Background:**

Phlegm-dampness constitution is a traditional Chinese medicine constitution typically associated with essential hypertension. Previous studies have demonstrated that auricular acupuncture effectively decreases blood pressure and adjusts the constitution. However, the mechanism underlying auricular acupuncture’s effect is poorly understood.

**Methods:**

A non-blinded, randomized controlled trial will be undertaken between September 2022 and May 2023. Eighty essential hypertensive patients with a phlegm-dampness constitution will be randomly allocated to one of two groups. The intervention group will receive eight weeks of auricular acupuncture and regular use of antihypertensive drugs, while the control group will only receive antihypertensive drugs. The primary outcome will be any mean differences in office systolic blood pressure. The secondary outcomes investigations will include proteins of the renin-angiotensin system, office blood pressure of different genotypes, and phlegm-dampness constitution scores.

**Discussion:**

By demonstrating how auricular acupuncture affects the renin-angiotensin system, this research will offer significant new information on the mechanism underlying the action of auricular acupuncture in hypertension. Moreover, the results will provide crucial clinical information on the associations between renin-angiotensin system gene polymorphisms and the antihypertensive effects of auricular acupuncture.

**Trial registration:**

Registered at the chictr.org.

## Introduction

Essential hypertension (EH) is a major contributor to cardiovascular disease and premature death worldwide [1, 2]. Effective blood pressure management is critical to reducing the morbidity and mortality of chronic diseases such as coronary heart disease and stroke [3]. EH is most prevalent among middle-aged and elderly individuals [4, 5]. Nearly half of Chinese people aged 35–75 years have EH; however, less than one-third receive treatment, and less than one-twelfth have their blood pressure under control [6].

Emerging evidence indicates that EH is closely associated with the traditional Chinese medicine (TCM) constitution [7, 8]. The TCM constitution is a new branch of TCM [9], essential in analyzing disease initiation, development, and prognosis and guiding disease prevention and treatment [10]. According to TCM principles, there are nine constitution types, among which a balanced constitution is a normal constitution, and the other eight are biased constitutions [11]. Phlegm-dampness constitution (PDC) is one of the most commonly biased constitutions [12]. People with PDC have a sluggish metabolic rate and irregular peripheral circulation. They are more likely to experience problems with lipid and energy metabolism [13]. A recent meta-analysis focusing on the association between the TCM constitution and hypertension in 12335 adults in China revealed that people with PDC were at considerable risk of developing hypertension (OR = 2.24, 95%CI: 1.77–2.83) [14]. Therefore, intervention measures for EH patients with PDC are clinically significant.

Non-pharmacological TCM therapies have been extensively used to treat EH [15]. As a time- and cost-efficient, relatively safe intervention, auricular acupuncture might be a promising treatment option [16]. Western medicine considers that auricular acupuncture can affect the internal organs through the nerve-body fluid channel. Thus, stimulating a particular ear point might regulate the endocrine system and enhance organ function [17]. Numerous clinical studies have been performed to determine whether auricular acupuncture is beneficial for treating hypertension [18]. However, the mechanism underlying the hypotensive effect of auricular acupuncture still requires clarification.

The renin-angiotensin system (RAS) has been shown to play a pathophysiological role in the development and progression of EH [19]. The classical RAS pathway is composed of angiotensin-converting enzyme (ACE), angiotensin Ⅱ (Ang Ⅱ), and angiotensin Ⅱ type 1 receptor (AT_1_R). In this pathway, ACE cleaves angiotensin Ⅰ to Ang Ⅱ. Then, Ang Ⅱ binds to AT_1_R to promote vasoconstriction and water and sodium retention [20]. A counter‐regulatory pathway, the ACE2‐Ang (1–7)‐Mas receptor (MasR) axis, is currently unfolding, which elicits protective actions, including vasodilation and increased nitric oxide synthesis [21]. Previous studies have confirmed that acupuncture can arrest the development of hypertension in spontaneously hypertensive rats by regulating ACE, AT_1_R, and angiotensin Ⅱ type 2 receptor (AT_2_R) [22, 23] and can improve quality of life by downregulating the ACE-AngII-AT_1_R axis and upregulating the ACE2-Ang (1–7)-MasR axis [24]. Thus, it can be hypothesized that the RAS may be related to the mechanism of auricular acupuncture.

Based on the characteristics of PDC and EH, an auricular acupuncture intervention scheme was formulated. This trial aims to explore the mechanism underlying the effect of auricular acupuncture on blood pressure. At the same time, with the help of genomics technologies, we will identify the relationships between RAS gene polymorphisms and the auricular acupuncture treatment effect. In addition, the effectiveness of the auricular acupuncture in regulating PDC will also be investigated.

## Materials and methods

### Study design

**Fig 1:**
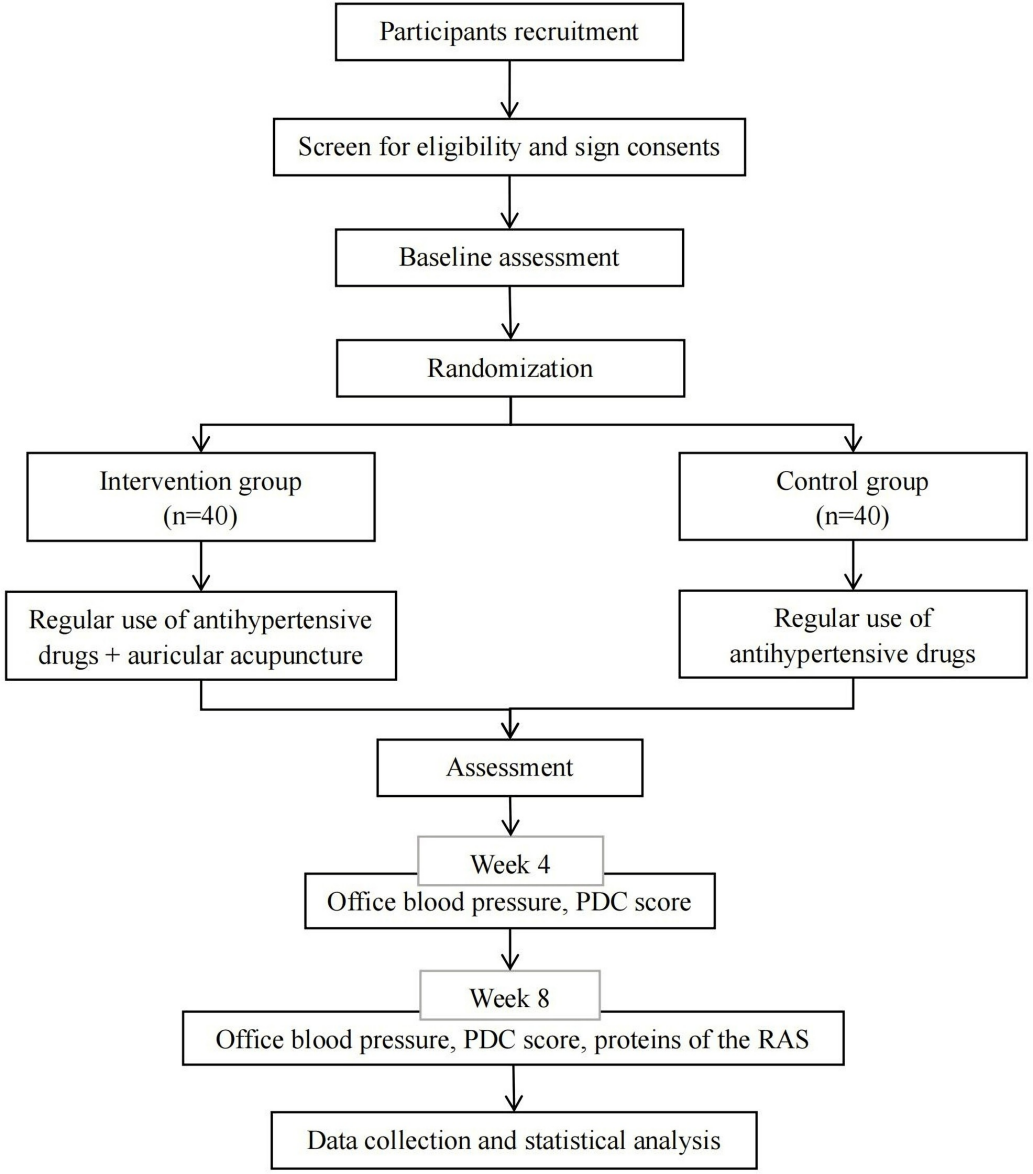
Flowchart of the trial design. Baseline assessment includes gene polymorphism testing (only intervention group), office blood pressure, PDC score, and proteins of the RAS. PDC, phlegm-dampness constitution; RAS, renin-angiotensin system.

This study is a non-blinded, randomized controlled trial (RCT) with two parallel groups. It will be performed at the Qingbo Street Community Health Service Center in Hangzhou, Zhejiang, China, between September 2022 and May 2023 (**Fig 1**).

### Ethics and dissemination

The present study protocol has been approved by the Regional Ethics Authority of the Zhejiang Chinese Medical University (20220310–3). It conforms to the Standard Protocol Items: Recommendations for Clinical Interventional Trials (SPIRIT) guidelines [25]. After the study procedures have been fully explained, the investigator will obtain and archive the informed consent forms from the participants that meet the eligibility criteria. Participant data will be recorded in CRFs. If the researchers need to get the data in the CRF, they must report to the primary investigator.

### Eligibility criteria

#### Inclusion criteria

(1) Individuals of both genders, aged between 45 and 74 years; (2) Diagnosed with PDC by tongue observation and the Constitution in Chinese Medicine Questionnaire [26] (**S1 Table**); (3) Diagnosed with EH according to the diagnostic criteria of the International Society of Hypertension Global Hypertension Practice Guidelines [27]; (4) The use of antihypertensive drugs limited to calcium channel blockers and the dosage is one capsule daily.

#### Exclusion criteria

(1) Individuals with mixed TCM constitutions; (2) Allergy to the medical devices involved in this trial; (3) Poor medication compliance; (4) The presence of serious complications such as severe arrhythmia or end-stage renal disease; (5) Ear infections, ulcers, swelling, or lack of skin integrity at the application sites during the course of the study; (6) Pregnancy or lactation during the trial; (7) History of mental disease; (8) Presence of malignant tumors; (9) Has received any other antihypertensive clinical test.

#### Intervention

Participants will be randomly divided into an intervention group and a control group. Besides regular antihypertensive drugs, the intervention group will receive an eight-week intervention with auricular acupuncture. The control group will only receive antihypertensive drugs.

The results of our previous study showed that combining antihypertensive drugs with an eight-week course of auricular acupuncture may be more effective in regulating both the yin deficiency constitution and blood pressure compared to using drugs alone [28]. Therefore, an eight-week auricular acupressure period will be implemented to improve the phlegm-dampness constitution and blood pressure in this trial.

**Fig 2:**
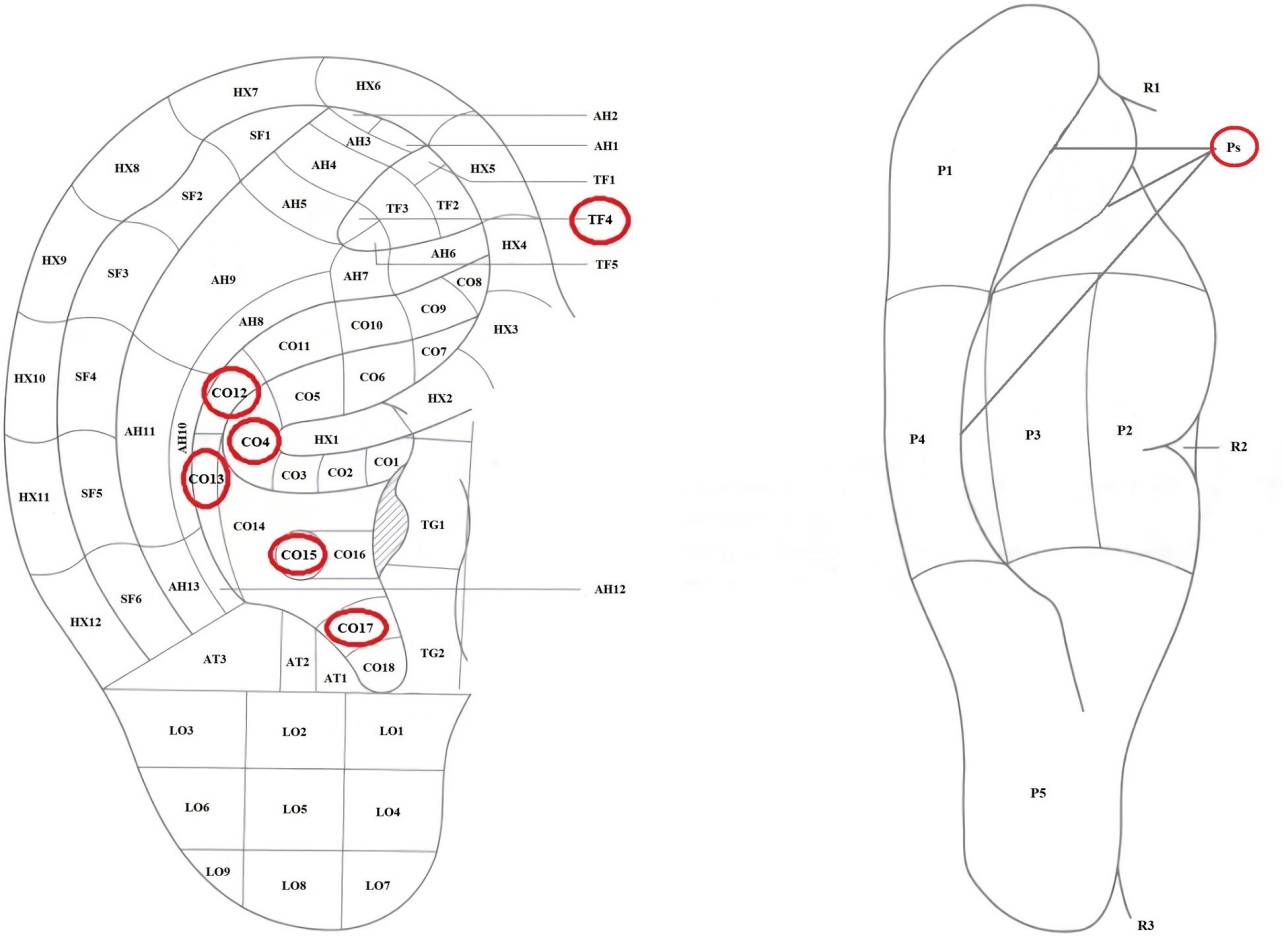
Auricular acupuncture points location (anterolateral and posteromedial). PS, Groove; TF_4_, Shenmen; CO_4_, Stomach; CO_12_, Liver; CO_13_, Spleen; CO_15_, Heart; CO_17_, Triple Energizer.

The acupoints selection scheme for auricular acupuncture, as described below, has been determined based on published literature and consultation with TCM experts [29–31]: Groove (P_S_), Shenmen (TF_4_), Stomach (CO_4_), Liver (CO_12_), Spleen (CO_13_), Heart (CO_15_), Triple Energizer (CO_17_). The names and locations of the auricular acupoints are based on the World Federation of Acupuncture Societies standard 002:2013 [32] (**Fig 2**). Detailed location and functional information on the auricular acupuncture points are presented in **S2 Table**.

The operational approach for auricular acupuncture will be as follows. First, participants will be seated, and the auricular acupoints’ skin will be sterilized using a 75% alcohol swab. A sterilized XS100-A acupoint detector will probe and mark each acupoint. An adhesive patch (0.5 cm*0.5 cm) with semen vaccariae will be taped on the points. Well-trained and certified auricular therapists will instruct all participants to apply pressure to each auricular acupoint using their thumb and index finger until they feel pain and heat, referred to as the “De-Qi” sensation. Participants will be supported to self-perform acupressure 20 to 30 times on each auricular acupoint. This self-performed acupressure should be done three times a day, in the morning, noon, and evening. Researchers will regularly remind the participants to do so using WeChat messages. Participants will be asked to record their everyday self-perform acupressure frequency. In the subsequent replacement of auricular plaster, researchers will check participants’ records and remind them to press the auricular point as required. Auricular acupuncture will be carried out on the auricular acupoints on one ear in one visit and the opposite ear in the following visit. The adhesive patch will be replaced by the researcher every three days over the eight-week duration of the trial.

#### Outcomes

The relevant time points of the study are listed in **S3 Table**.

#### Primary outcome

*Office systolic blood pressure*. The primary outcome of this study will be the difference in mean office systolic blood pressure. Both groups will utilize a certified electronic sphygmomanometer (HBP-1300, Omron Corporation, Japan) to assess blood pressure levels in the morning at fourth and eighth weeks. Patients will be asked to rest for at least ten minutes and then measure their right upper arm blood pressure in a sitting position, with the right upper arm at heart level [33]. Three consecutive office blood pressure measurements will be performed at three-minute intervals, and the average of the second and third readings will be recorded. The mean office systolic blood pressure differences between the intervention and control groups will be analyzed at fourth and eighth weeks.

#### Secondary outcomes

*Proteins of the RAS*. The blood sample will be collected from each eligible participant at baseline and eighth weeks after allocation. The blood sampling time was between 8 to 9 in the morning. Serum concentrations of ACE, AngⅡ, ACE2, and Ang (1–7) will be detected using enzyme-linked immunosorbent assay. The participants must avoid drinking alcohol and intense exercise one day before sample collection. To avoid scheduling blood collection during the menstrual period of female participants, their menstrual cycles will be asked during the enrollment process. The serum will be separated from the blood using centrifugation and stored at -80°C until final analysis. The difference in mean change of proteins from baseline to 8 weeks may reveal the mechanism of auricular acupuncture.

*Office blood pressure of different genotypes*. Only participants in the intervention group will have gene polymorphism testing at baseline. Five single nucleotide polymorphisms (ACE, ACE2, AT_1_R, AT_2_R, Mas) will be genotyped using mass spectrometry. The changes in office blood pressure of different genotypes will be analyzed to determine whether genotype influences the blood pressure-decreasing response to antihypertensive treatment with auricular acupuncture.

*PDC score*. Patients’ PDC scores will be determined by TCM physicians using the Constitution in Chinese Medicine Questionnaire compiled by Professor Wang Qi in 2009 [26]. This scale consists of nine subscales. The original score of the PDC subscale is equal to the sum of the scores for each item, and the transformed score is calculated as follows: [(original score—number of items) / (number of items × 4)] ×100. The transformed score of the PDC subscale ranges from 0–100. PDC score will be measured at baseline, fourth and eighth weeks after allocation, and the transformed score will be recorded.

#### Sample size calculation

The sample size for this RCT has been computed based on a previous study [34]. In previous study, auricular acupuncture was associated with a further 8 mmHg (δ) of office systolic blood pressure reduction compared with the control group. The combined standard deviation was 10.4 (σ). The sample size was determined using a superiority test in R programming language (version 4.2.1). Setting a significance level (α) of 0.05, a power (β) of 0.10, and a superiority margin of 0.10. A dropout rate of 10% is anticipated, and 40 participants per group will be enrolled in the study for a total required sample size of 80.

#### Recruitment

Bulletin board advertisements will be posted, and community doctors will be contacted to recruit participants. The recruitment information will primarily include the study eligibility criteria and researcher contact details. A well-trained investigator will be responsible for the recruitment of participants. The recruitment will be conducted at Qingbo Street Community Health Service Center between September 2022 and March 2023.

#### Randomization

A statistician will generate a random sequence using SPSS 25.0 software. The random sequence will be created using a random block size of 4 with a 1:1 allocation and placed in opaque envelopes. Eligible participants will receive an envelope in the order of enrollment and will deliver this to the auricular therapist. The researcher who sealed the random sequence envelopes will not participate in the participants’ inclusion, treatment, or evaluation.

#### Blinding

Due to the nature of auricular acupuncture, the participants and auricular therapists cannot be blinded. However, the participants will be treated individually, and the auricular therapists will not be engaged in any aspect of outcome assessment. The assessor and the statistician will be blinded to the group allocation.

#### Data management and monitoring

Before data collection, all researchers will be trained uniformly to ensure data quality. Two well-trained researchers will enter the data into password-protected computer software (Excel^TM^ Microsoft, 2021) with double entry and range checks. Any modifications will be noted with an apparent reason and will need to be signed and dated. A Data Monitoring Committee will not be established because of the short duration and minimal risks.

### Statistical analysis

The statistician will use SPSS 25.0 software to analyze the data; a *P*-value < 0.05 will be considered statistically significant. Continuous variables will be expressed as means and standard deviations or medians and interquartile ranges. Categorical variables will be expressed as frequencies and percentages. The mean office blood pressure value, PDC score, and protein content are continuous variables. The difference in those outcomes between groups at baseline, fourth and eighth weeks and the difference before and after treatment in each group will be analyzed using repeated measures ANOVA or mixed linear regression analyses. Both intention-to-treat (ITT) population and per-protocol (PP) population will be applied in the data analysis. The outcome of the ITT analysis will be compared to that of the PP analysis to ascertain whether the outcomes are consistent. If data is missing, the last value will be used for interpolation. A safety analysis will be used for safety evaluation. In addition, a subgroup analysis will be performed based on age (45–65 and 66–75) to separate the mechanism of auricular acupuncture.

### Safety assessment

Although auricular acupuncture is typically considered a safe treatment, participants will be assessed for discomfort. Details of all adverse events will be recorded during the trial in the case report forms (CRFs). The ethics committee will examine any connections between adverse events and the intervention and decide whether the study should continue. The occurrence rate of adverse events will be calculated as (number of cases with adverse events / total number of cases) × 100% to evaluate the safety of auricular acupuncture.

## Discussion

Auricular acupuncture is one of the most commonly used non-pharmacological TCM therapies in the clinical treatment of EH. It has a huge potential market demand and broad application prospects. However, our understanding of the biological foundation of auricular acupuncture is limited. Even though metabolic dysregulation of the RAS has been closely linked to the pathogenesis of EH [20], few studies have evaluated protein changes in the RAS following auricular acupuncture treatment. This study incorporates immunomics technology to characterize the protein signatures associated with changes in clinical outcomes. This study will offer critical physiological details to comprehend the mechanism underlying the effect of auricular acupuncture on blood pressure. It will provide clinical information to guide the non-pharmacologic management of EH.

Additionally, differences in the changes in office blood pressure between different genotypes will further reveal the associations between RAS gene polymorphisms and the antihypertensive effect of auricular acupuncture in patients with PDC. These data may provide evidence to inform precision non-pharmacologic blood pressure management.

Moreover, PDC is not only implicated in the occurrence and development of EH but also plays a crucial role in determining the prognosis of EH [35]. Therefore, regulating PDC is an indispensable part of hypertension management. However, few pharmacological therapies aim to change the TCM constitution. Based on the “recuperability of constitution” concept, the acupoints selected in this trial are targeted at decreasing blood pressure, expelling phlegm, and dispelling dampness. The current study’s PDC score change will demonstrate that auricular acupuncture can alter PDC [9].

However, there are some limitations in this study. A placebo control group will not be set in this study since a placebo in non-pharmacological trials is challenging. Therefore, the placebo effect on blood pressure and RAS cannot be ruled out. The study also has a limitation in that blinding could not be implemented. Additionally, considering follow-up was not planned for in the trial, how long the potential benefit of auricular acupuncture persisted could not be ascertained.

## Supporting information

S1 Checklist SPIRIT checklist with page reference numbers. (DOC)

S1 Table The measuring scale for phlegm-dampness constitution of traditional Chinese medicine. The original score is equal to the sum of the scores for each item, and the transformed score is calculated as follows: [(original score—number of items)/(number of items × 4)] ×100. When the transformed score ≥ 40, it is phlegm-dampness constitution; scored 30 to 39, it tend to be phlegm-dampness constitution; < 30, it was not phlegm-dampness constitution.
(DOCX)

S2 Table Detailed location and function information on auricular acupuncture points. (DOCX)

S3 Table Trial process chart. (DOCX)

S1 File Trial protocol for ethics application. (DOCX)

S2 File (DOCX)
