# Supplementary material for: Renin-angiotensin system mechanism underlying the effect of auricular acupuncture on blood pressure in hypertensive patients with phlegm-dampness constitution: Study protocol for a randomized controlled trial
Source: PLoS One. 2024 Feb 1;19(2):e0294306. doi: 10.1371/journal.pone.0294306 (PMC10833565; doi:10.1371/journal.pone.0294306)
Supplement: S1 Table — The original score is equal to the sum of the scores for each item, and the transformed score is calculated as follows: [(original score—number of items)/(number of items × 4)] ×100. When the transformed score ≥ 40, it is phlegm-dampness constitution; scored 30 to 39, it tend to be phlegm-dampness constitution; < 30, it was not phlegm-dampness constitution. (DOCX) [file pone.0294306.s002.docx]

**Supplemental table 1 The measuring scale for phlegm-dampness constitution of traditional Chinese medicine**

| **Experience/condition in the Past Year** | **No** | **Slightly** | **Sometimes** | **Often** | **All the time** |
| --- | --- | --- | --- | --- | --- |
| 1. **Does your body feel heavy or lethargic?** | 1 | 2 | 3 | 4 | 5 |
| 1. **Do you feel chest or stomach stuffiness?** | 1 | 2 | 3 | 4 | 5 |
| 1. **Do you have oily skin on your forehead?** | 1 | 2 | 3 | 4 | 5 |
| 1. **Do you upper eyelids swell larger than others (slight swelling at the upper eyelid)?** | 1 | 2 | 3 | 4 | 5 |
| 1. **Does your mouth feel sticky?** | 1 | 2 | 3 | 4 | 5 |
| 1. **Is your stomach/belly flabby?** | 1 | 2 | 3 | 4 | 5 |
| 1. **Do you always have lots of phlegm, especially in your throat?** | 1 | 2 | 3 | 4 | 5 |
| 1. **Does your tongue have a thick coating?** | 1 | 2 | 3 | 4 | 5 |

The original score is equal to the sum of the scores for each item, and the transformed score is calculated as follows: [(original score - number of items)/(number of items × 4)] ×100. When the transformed score ≥40, it is phlegm-dampness constitution; scored 30 to 39, it tend to be phlegm-dampness constitution; ＜30, it was not phlegm-dampness constitution.
