# Supplementary material for: Renin-angiotensin system mechanism underlying the effect of auricular acupuncture on blood pressure in hypertensive patients with phlegm-dampness constitution: Study protocol for a randomized controlled trial
Source: PLoS One. 2024 Feb 1;19(2):e0294306. doi: 10.1371/journal.pone.0294306 (PMC10833565; doi:10.1371/journal.pone.0294306)
Supplement: S2 Table — (DOCX) [file pone.0294306.s003.docx]

**Supplemental table 2 Detailed location and function information on auricular acupuncture points**

| **Name** | **Location** | **Function** |
| --- | --- | --- |
| **Groove (P_S_)** | On the posteromedial surface of the ear formed by the superior and inferior antihelix crura | Can relax and decrease blood pressure. It is a key acupoint for treating hypertension. |
| **Shenmen (TF_4_)** | In the upper part of the posterior 1/3 of the triangular fossa | Can relax and decrease blood pressure. It is a key acupoint for treating hypertension. |
| **Stomach (CO_4_)** | At the end of the helix crus | Can regelate stomach-energy and lower the adverse rising energy, promote digestion and resolve phlegm. Stimulation on it can regulate the phlegm-dampness constitution. |
| **Liver (CO_12_)** | In the posterosuperior part of the cymba conchae | Can sooth liver to regulate Qi, remove dampness and activating blood. Stimulation on it can regulate the phlegm-dampness constitution. |
| **Spleen (CO_13_)** | In the region inferior to line BD, posterosuperior to the cavum conchae | Can transport and transform nutrients from foodstuff, strengthen the spleen and ourish Qi, remove phlegm and resolve turbid pathogens. Stimulation on it can regulate the phlegm-dampness constitution. |
| **Heart (CO_15_)** | In the center of the depression of the cavum conchae | Can relieve mental stress, harmonic nutrient blood, and reduce internal heat. It is a key acupoint for treating cardiovascular and cerebrovascular diseases. |
| **Triple Energizer (CO_17_)** | In the region posteroinferior to the orifice of the external auditory meatus | Can dredge and regulate water passage, remove phlegm and resolve turbid pathogens. Stimulation on it can regulate the phlegm-dampness constitution. |
