# Supplementary material for: Renin-angiotensin system mechanism underlying the effect of auricular acupuncture on blood pressure in hypertensive patients with phlegm-dampness constitution: Study protocol for a randomized controlled trial
Source: PLoS One. 2024 Feb 1;19(2):e0294306. doi: 10.1371/journal.pone.0294306 (PMC10833565; doi:10.1371/journal.pone.0294306)
Supplement: S3 Table — (DOCX) [file pone.0294306.s004.docx]

**S3 Table Trial process chart**

|  | **Enrolment** | **Allocation** | **Post-allocation** | | | |
| --- | --- | --- | --- | --- | --- | --- |
| **TIMEPOINT** | **Week 0** | | **Week 2** | **Week 4** | **Week 6** | **Week 8** |
| **ENROLMENT:** |  |  |  |  |  |  |
| Eligibility screen | √ |  |  |  |  |  |
| Informed consent | √ |  |  |  |  |  |
| Demographics | √ |  |  |  |  |  |
| Medical history | √ |  |  |  |  |  |
| Allocation |  | √ |  |  |  |  |
| **INTERVENTIONS:** |  |  |  |  |  |  |
| Intervention group |  |  |  |  |  |  |
| Control group |  |  |  |  |  |  |
| **ASSESSMENTS:** |  |  |  |  |  |  |
| Office blood pressure |  | √ |  | √ |  | √ |
| PDC score |  | √ |  | √ |  | √ |
| Proteins of the RAS |  | √ |  |  |  | √ |
| **PARTICIPANTS’ SAFETY:** |  |  |  |  |  |  |
| Adverse event |  |  |  |  |  |  |
| Safety assessment |  |  |  |  |  |  |
| Causes of dropout |  |  |  | √ |  | √ |
| Compliance analysis |  |  |  | √ |  | √ |
| Demographics include age, gender, height, weight, body mass index, waist/hip circumference, smoking history, and drinking history. Medical history includes hypertension duration, name of antihypertensive drugs, other diseases, other medications, and drug compliance. PDC, phlegm-dampness constitution; RAS, renin-angiotensin system. | | | | | | |
