# Supplementary material for: Renin-angiotensin system mechanism underlying the effect of auricular acupuncture on blood pressure in hypertensive patients with phlegm-dampness constitution: Study protocol for a randomized controlled trial
Source: PLoS One. 2024 Feb 1;19(2):e0294306. doi: 10.1371/journal.pone.0294306 (PMC10833565; doi:10.1371/journal.pone.0294306)
Supplement: S1 File — (DOCX) [file pone.0294306.s005.docx]

**1. Declaration**

The study is conducted in strict accordance with the project plan and records the test data truthfully and accurately; there is no conflict of interest.

**2. Research title**

Renin-angiotensin system mechanism underlying the effect of auricular acupuncture on blood pressure in hypertensive patients with phlegm-dampness constitution

**3. Funding**

Zhejiang province scientific research fund project of traditional Chinese medicine (2022ZA053)

**4. Trial process chart**

| Outcomes | Evaluation time | | |
| --- | --- | --- | --- |
|  | Baseline | 4 weeks after allocation | 8 weeks after allocation |
| Blood pressure | × | × | × |
| phlegm-dampness constitution score | × | × | × |
| Proteins of the RAS | × |  | × |
| Gene polymorphism testing (only intervention group) | × |  |  |

**5. Background**

Essential Hypertension (EH) is a major contributor to the cardiovascular disease and premature death worldwide. It is also a serious disease that seriously endangers human health. Effective management of blood pressure is a key means to reduce the morbidity and mortality of chronic diseases such as coronary heart disease and stroke.EH is most prevalent among middle-aged and elderly individuals. Nearly half of Chinese people aged 35-75 years have EH; however, less than one-third receive treatment and less than one-twelfth have their blood pressure under control. Therefore, the prevention and treatment of EH in this group has become one of the important problems to be solved in the medical community today. Precision medicine proposed by Western medicine, also known as individualized treatment, helps patients, with the help of cutting-edge technologies such as genomic, proteomics determination and living environment, to develop optimal treatment plans for individual disease characteristics, in order to pursue the maximum treatment effect and the lowest side effects. This is similar to the idea of “preventivetreatment of disease” and the idea of “Dialectical treatment” in the traditional Chinese medicine (TCM) constitution.

**1. The TCM constitution is the expression of the basic elements of precision medicine, TCM constitution can be the “Chinese-style precision medicine”**

Emerging evidence indicates that EH is closely associated with the TCM constitution. The TCM constitution is a new branch of TCM; it plays an important role in analyzing the initiation, development, and prognosis of disease, and in guiding disease prevention and treatment. According to TCM principles, the TCM constitution depends on the intrinsic characteristics of the human body and is affected by the environment. There are nine constitution types including balanced constitution, qi-deficiency constitution, yang-deficiency constitution, yindeficiency constitution, phlegm-dampness constitution, dampness-heat constitution, blood stasis constitution, qi stagnation constitution, and inherited special constitution (balanced constitution is a normal constitution, and the other eight types are biased constitutions). Through analysis of the literature, we found that phlegm-dampness constitution (PDC) is one of the most common biased constitution in EH patients, with an incidence of 14.62% to 43.0%. A recent meta-analysis revealed that people with PDC were at considerable risk of developing hypertension. Therefore, intervention measures for EH patients with PDC are of great clinical significance.

**2. Compared with pharmacological treatment, TCM non-pharmacological treatment also plays an important role in the prevention and treatment of EH**

EH can be treated by both pharmacological and non-pharmacological approaches. Pharmacological trials have shown that only 25% to 62% of EH patients can reduce blood pressure and maintain it in the normal range by monotherapy, and most patients need more than one antihypertensive drug, which will increase the economic burden of patients and increase the risk of drug side effects. Therefore, choosing inexpensive, safe and easy to implement treatments is crucial for the prevention and treatment of hypertension. TCM non-pharmacological treatments are safe and easy, and only need short-term training, suitable for EH patients. As a time- and cost-efficient intervention that is relatively safe, auricular acupuncture might be a promising treatment option. TCM theory holds that diseases are caused by an imbalance in a person’s Qi and that the ears are directly or indirectly connected with the 12 meridians; thus, stimulating the corresponding acupoints in the ear can help to correct this imbalance. Auricular acupuncture restores the body to harmony in a gentle way and helps to solve health problems. Based on the characteristics of PDC and EH, the auricular acupuncture intervention scheme was formulated. To lower the blood pressure and improve the TCM constitution of EH patients with PDC can reflect the “Chinese-style precision medicine”. Numerous clinical studies have been performed to determine whether auricular acupuncture is beneficial for the treatment of hypertension. However, the mechanism underlying the hypotensive effect of auricular acupuncture still requires clarification.

**3. The RAS may be related to the mechanism of auricular acupuncture, The mechanism maybe involve ACE, AngⅡ, AT_1_R, ACE2, Ang (1-7), MasR**

The renin-angiotensin system (RAS) has been shown to play a pathophysiological role in the development and progression of EH. The classical RAS pathway is composed of angiotensin-converting enzyme (ACE), angiotensin Ⅱ (Ang Ⅱ), and angiotensin Ⅱ type 1 receptor (AT_1_R). In recent years, the new members of the RAS system, angiotensin-converting enzyme 2 (ACE2), angiotensin (1-7) [Ang (1-7)] and its receptor (MasR), constitute the new axis of RAS, namely, ACE2-Ang (1-7) -MasR axis.

In the ACE-Ang II-AT_1_R axis, renin enzymatically cleaves angiotensinogen to Ang Ⅰ, which is subsequently cleaved by ACE to form Ang Ⅱ. Then, Ang Ⅱ binds to AT_1_R to promote vasoconstriction and water and sodium retention. It can also bind to angiotensin Ⅱ type 2 receptor (AT_2_R) to promote hypotensive effects.

In the new axis of RAS, ACE2 cleaves Ang II to generate Ang (1-7), or competes with ACE for the catalytic common substrate Ang I to generate Ang (1-9), Ang (1-9) and Ang (1-7) through ACE, and the main receptor of Ang (1-7) is Mas. Ang (1-7) can exert its antihypertensive effect in the following three ways: (1) reduce AngⅡ production by inhibiting ACE; (2) release vasodilation factors such as bradykinin, nitric oxide, prostaglandin and endothelial hyperpolarization factors to dilate blood vessels, inhibit cardiovascular remodeling and resist oxidative stress; (3) increase the release of prostacyclin I_2_ and cyclic adenylate production, achieving the regulation of blood pressure.

Previous studies have confirmed that acupuncture can arrest the development of hypertension in spontaneously hypertensive rats by regulating ACE, AT_1_R, and AT_2_R and can improve the quality of life by downregulating the ACE-Ang II-AT_1_R axis and upregulating the ACE2-Ang (1-7)-MasR axis. In addition, one study have shown that auricular acupuncture may lower the blood pressure of EH patients with yin-deficiency constitution by up-regulating ACE protein and down-regulating Ang (1-7) protein, therefore, we hypothesized that the RAS may be related to the mechanism of auricular acupuncture, and further speculate that auricular acupuncture may can regulating the protein of ACE, AngⅡ, ACE2, and Ang (1-7).

**4. Genotypes may affect the sensitivity of EH patients with PDC to auricular acupuncture.**

By summarizing the existing clinical research literature, it have found that auricular acupuncture can reduce blood pressure and improve related symptoms, but some patients are not sensitive to auricular acupuncture in many researches. Furthermore, multiple studies have shown that antihypertensive drug efficacy is associated with the gene polymorphism of RAS. Huang Hong’s study showed that In EH patients with different genotypes of ACE gene, the antihypertensive efficacy of irbesartan and the reduction of plasma RAS activity before and after irbesartan treatment were different, manifested as DD > ID > II, which shows that the ACE I/D polymorphism may be an important indicator affecting the antihypertensive response to irbesartan in EH patients. Gong Hongtao using polymerase chain reaction to analyze AT_1_R genes 1166A/C, -810A/T and -521C/T polymorphism of the patients, The results showed a significant difference in the comparison of diastolic blood pressure drop before and after using telmisartan between the AT_1_R gene -521C/T CC genotype and CT + TT genotype (P<0.05), it showed that the -521C/T polymorphism of AT_1_R gene can independently predict the individual difference in patients' response to telmisartan. Men Chen investigated the relationship of A1675G gene polymorphism of AT_2_R and antihypertensive efficacy of losartan in EH patients, found that A allele may be the key factor influencing the antihypertensive efficacy of losartan. In conclusion, gene polymorphisms determine the differences in the effect of antihypertensive drugs, resulting in individual differences in the effect of drug treatment. Therefore, it is speculated that gene polymorphisms may also affect the effect of auricular acupuncture, but no relevant study has reported the relationship between the effect of EH and auricular acupuncture in patients with different genotypes. This study intends to test the polymorphisms of key genes of RAS in EH patients with PDC, analyze the sensitivity of different genotypes and alleles to auricular acupuncture, and provide a reference for improving the efficacy of auricular acupuncture.

**6. Research purposes**

(1) The changes of proteins of the RAS before and after auricular acupuncture will be compared to explore the mechanism underlying the effect of auricular acupuncture on blood pressure.

(2) Participants in the intervention group will have gene polymorphism testing, the changes in blood pressure of different genotypes will be analyzed to determine whether genotype influence the blood pressure-decreasing response to antihypertensive treatment with auricular acupuncture. And to identify genes of RAS that are sensitive to auricular acupuncture.

**7. Inclusion/Exclusion criteria**

**Inclusion criteria:**

- Individuals of both sexes, aged between 45 and 74 years;
- diagnosed with PDC;
- diagnosed with EH according to the diagnostic criteria of the International Society of Hypertension Global Hypertension Practice Guidelines.

**Exclusion criteria:**

- Individuals with mixed TCM constitutions;
- allergy to the medical devices involved in this trial;
- ear infections at the application sites during the course of the study;
- taking ACE inhibitors or angiotensin receptor blockers;
- poor medication compliance;
- the presence of serious complications such as severe arrhythmia or end-stage renal disease;
- pregnancy or lactation during the trial;
- history of mental disease;
- presence of malignant tumors;
- has received any other antihypertensive clinical test.

**8. Study design**

Randomized controlled trial, parallel


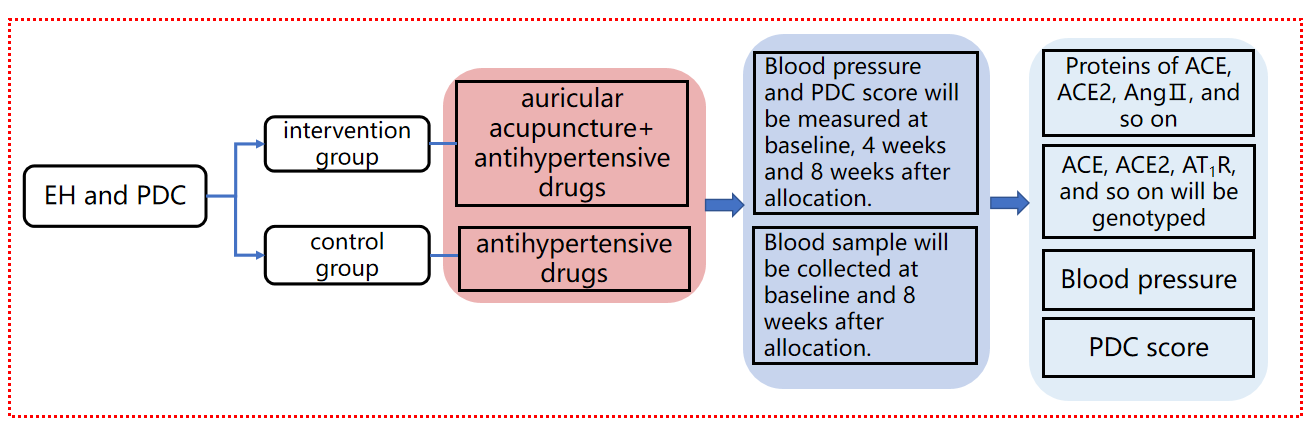


**9. Sample size calculation**

The sample size for this RCT has been computed based on our previous study (36). In our previous study, the efficacy rate was 28% in the intervention group and 0% in the control group after eight weeks of auricular acupuncture intervention. Setting an α-value of 0.05 and a β-value of 0.10, the sample size was calculated using the following formula:

$$n=\frac{{2\bar{p}\bar{q}\left( z_{\alpha}+z_{\beta} \right)}^{2}}{\left( p_{1}-p_{2} \right)^{2}}$$

Considering a dropout rate of 20% during the research, a total of 40 participants per group will be enrolled in the study, for a total required sample size of 80.

**10. Randomization**

A statistician will generate a random sequence using SPSS 25.0 software and the random sequence will be placed in opaque envelopes. Eligible participants will receive an envelope in the order of enrollment and will deliver this to the auricular therapist. The researcher who sealed the random sequence envelopes will not take part in the inclusion, treatment, or evaluation of the participants.

**11. Blinding**

The participants will be treated individually, and the assessor and the statistician will be blinded to the group allocation.

**12. Outcomes**

**Main outcome**

**Efficacy of auricular acupuncture:** The blood pressure will be measured at baseline, 4 weeks and 8 weeks after allocation. The efficacy of auricular acupuncture is defined as office diastolic blood pressure is reduced to a threshold set out in “Guiding Principles for Clinical Research of New Chinese Medicines” formulated by the Ministry of Health of the Peoples Republic of China a significant effect means a diastolic blood pressure decrease ≥10 mmHg and return to normal level or a decrease ≥ 20 mmHg. An effect means a diastolic blood pressure decrease <10 mmHg and return to normal level or decrease of 10 to 20 mmHg. Invalid means that the change in blood pressure did not reach the “effect” level described above.

Requirements for blood pressure measurement:

(1) blood pressure will be measured in the afternoon and in a quiet room with comfortable temperature (22℃-24℃) by one well-trained researcher.

(2) use validated upper-arm cuff device and sphygmomanometer for blood pressure measurement, and ensure the calibration before the measurement.

(3) Before measurements: remain seated and relaxed for at least 5 min; avoid smoking, caffeine and exercise for 30 min; empty bladder.

(4) Sit in a backrest chair with back supported on chair, legs uncrossed and feet flat on floor, arm resting on table with mid-arm at heart level.

(5) Tie the cuff to the upper arm, and tighten it so that it can be inserted 1 to 2 fingers. The lower edge of the cuff should be 2.5cm above the elbow fossa.

(6) Blood pressure of both upper arms is measured at first visit and the higher side is usually measured later. At each visit take 2 measurements with 1-2 min between them. Calculate the average of the 2 measurements. If the 2 readings of systolic or diastolic blood pressure varied by more than 10mmHg, the third time is taken and the average of the last two measurements is recorded.

**Secondary outcomes**

(1) **PDC score:** Patients’ PDC score will be determined by TCM physicians using the TCM Constitution Scale compiled by Professor Wang Qi in 2009. This scale consists of nine subscales. The original score of the PDC subscale is equal to the sum of the scores for each item, and the transformed score is calculated as follows: [(original score - number of items)/(number of items × 4)] ×100. PDC score will be measured at baseline, 4 weeks and 8 weeks after allocation, and the transformed score will be recorded.

(2) **Proteins of the RAS** **ACE、AngⅡ、ACE2、Ang (1-7):** Serum concentrations of ACE, AngⅡ, ACE2, Ang (1-7) will be tested using enzyme-linked immunosorbent assay. The participants will be required to avoid drinking alcohol and intense exercising one day before sample collection. Females should avoid having their menstrual period during collection. Serum will be separated from the blood by centrifugation and will be stored at -80℃ until final analysis. Blood sample will be collected from each eligible participant at baseline and 8 weeks after allocation.

**(3) Office blood pressure of different genotypes:** Only participants in the intervention group will have gene polymorphism testing at baseline. Five single nucleotide polymorphisms (ACE, ACE2, AT1R, AT2R, Mas) will be genotyped by mass spectrometry or polymerase chain reaction. The changes in office blood pressure of different genotypes will be analyzed to determine whether genotype influence the blood pressure-decreasing response to antihypertensive treatment with auricular acupuncture.

**13. Safety assessment**

Although auricular acupuncture is typically regarded as a safe treatment, participants will be assessed for any discomfort. Details of all adverse events will be recorded during the trial in the case report forms (CRFs). The ethics committee will examine any connections between adverse events and the intervention and make a decision on whether the study should be continued.

**14. Recruitment**

Bulletin board advertisements will be posted, and community doctors will be contacted to recruit participants. The recruitment information will mainly include the study eligibility criteria and researcher contact details. A well-trained investigator will be responsible for the recruitment of participants. The recruitment will be conducted at Qingbo Street Community Health Service Center from September 2022 to February 2023. After the study procedures have been fully explained, the investigator will obtain and archive the informed consent forms from the participants that meet the eligibility criteria.

**15. General information of the participants**

Chronological age; height; waist/hip circumference; gender; occupation; weight; body mass index (BMI); blood pressure (SBP/DBP); past history; family history; medications (The current drug use of patients for hypertension and other diseases).

**16. Baseline indicators and observation items**

Demographics include age, gender, height, weight, body mass index, waist/hip circumference, smoking history, and drinking history.

**17. Operational approach**

Participants will be seated and the skin on the auricular acupoints will be sterilized with a 75% alcohol swab. A sterilized XS100-A acupoint detector will be used to probe and mark each acupoint. An adhesive patch (0.5 cm*0.5 cm) with Wangbuliuxing seeds will be taped on the points. Well-trained and certified auricular therapists will instruct all participants to apply pressure to each auricular acupoint using their thumb and index finger until they feel pain and heat. Participants will be supported to self-perform acupressure 20 to 30 times on each auricular acupoint, three times daily. Auricular acupuncture will be carried out on the auricular acupoints on one ear in one visit and the opposite ear in the following visit. The adhesive patch will be replaced by the researcher every three days over the eight-week duration of the trial.

**18. Statistical analysis**

The statistician will use SPSS 25.0 software to analyze the data; a *P*-value < 0.05 will be considered statistically significant. Continuous variables will be expressed as means and standard deviations, or as medians and interquartile ranges. Categorical variables will be expressed as frequencies. Continuous variables will be analyzed by *t* tests or Wilcoxon rank-sum tests. The difference before and after treatment in each group will be determined using a paired *t* test. A chi-squared or Fisher’s exact test will be used to compare the efficiency between the intervention and control groups.

**19. Participants’ management**

- Record the participants’ name, contact information and other information, and keep the information confidential.
- Add the participants’ WeChat, put intervention group into the group chat, and remind them to press the ear point every day.
- Before the trial, the investigator fully informed the participants, respected their wishes and signed the informed consent.

**20. Sample management**

- Blood samples from each participants will be labeled and stored in a 4℃ ice box.
- Store in the -80℃ refrigerator after transportation to the laboratory.

**21. Data management**

Before data collection, all researchers will be trained uniformly to ensure data quality. Participant data will be recorded in CRFs. If the researchers need to alter the data in the CRF, they will need to report to the primary researcher. Any modifications will be noted with a clear reason and will need to be signed and dated. Two well-trained researchers will enter the data into password-protected Excel with double entry and range checks.

**22. Availability of data and materials**

After the graduate degree thesis is published, you can contact the supervisor for acquisition.

**23. Treatment and management of the participants after the trial**

Participants in the control group who did not receive auricular acupuncture will be treated by a free 2-month auricular acupuncture service.
