# Supplementary material for: Renin-angiotensin system mechanism underlying the effect of auricular acupuncture on blood pressure in hypertensive patients with phlegm-dampness constitution: Study protocol for a randomized controlled trial
Source: PLoS One. 2024 Feb 1;19(2):e0294306. doi: 10.1371/journal.pone.0294306 (PMC10833565; doi:10.1371/journal.pone.0294306)
Supplement: S2 File — (DOCX) [file pone.0294306.s006.docx]

**一、诚信申明**

本研究操作过程均严格按照课题方案进行，真实准确记录试验数据；不存在任何利益冲突。

**二、研究题目**

基于RAS双轴探讨耳穴贴压对高血压痰湿质患者调体降压的作用机制

**三、经费来源**

浙江省中医药科学研究基金（2022ZA053）

**四、研究事项执行流程表**

| 结局指标 | 评估时间 | | |
| --- | --- | --- | --- |
|  | 基线 | 干预后4周 | 干预后8周 |
| 血压 | × | × | × |
| 痰湿质评分 | × | × | × |
| RAS轴蛋白产物 | × |  | × |
| RAS轴关键基因（仅干预组） | × |  |  |

**五、研究背景**

原发性高血压又称高血压（Essential Hypertension，EH），是全球心血管疾病和过早死亡的主要危险因素，也是严重危害人类的健康的一类重大疾病，有效管理血压是降低冠心病和脑中风等慢性疾病发病率和死亡率的关键措施。近年来，中老年人已成为EH的主要人群，在35~75岁的中国人中，近一半患有EH，然而，不到1/3的EH患者正在接受治疗，不到1/12的血压得到控制。因此，对这一群体EH的防治已成为当今医学界需要解决的重要问题之一。西医提出的精准医学又称个体化治疗，即借助基因组、蛋白质组测定等前沿技术，结合患者生活方式和生活环境，为患者制定针对个体疾病特征的最优化治疗方案，以追求最大的治疗效果和最低的副作用。这与中医体质学说中的“治未病”思想以及“辨证论治”的理念有异曲同工之妙。

**1. 中医体质学说是对西医个体化治疗基本要素的中医表达，体质学说也成为“中国式的个体化治疗”**

最近研究证实EH与中医体质密切相关。中医体质是中医的一个新分支；它在分析疾病的发生、发展和预后以及指导疾病的预防和治疗方面发挥着重要作用。根据中医的基本原理，中医体质取决于人体的内在特征，并受到环境的影响。目前中医主要将人群体质分为九种，即平和质、阴虚质、气虚质、阳虚质、痰湿质、湿热质、血瘀质、气郁质和特禀质（平和质为正常体质，其余8种体质为偏颇体质）。中医体质可用于指导EH个体化防治，通过对文献整理和分析发现痰湿质是EH患者最常见的偏颇体质之一，发生率为14.62%~43.0%，同时Meta分析以及一项中国的大型回顾性研究也显示痰湿质是EH的易感体质。因此，对EH痰湿质患者采取中医个体化防治具有重要的临床意义。

**2. 与药物治疗相比，中医非药物治疗方法在EH防治中也起着重要的作用**

EH可以通过药物和非药物方法进行治疗。药理试验表明，只有25%~62%的EH患者可通过单药降低血压并维持在正常范围内，多数患者需要使用一种以上的抗EH药物进行治疗，这将增加患者的经济负担并加大药物副作用的发生风险。因此，选择价格低廉、安全且易于实施的治疗手段对于防治高血压至关重要。耳穴贴压，又称耳穴压豆，是一种传统中医非药物疗法，在EH防治中起着重要的作用。中医认为人是由“气”构成，气乱则病，而耳直接或间接与十二经脉相连，通过刺激耳部相应穴位可调节气机，恢复气血平衡。耳穴贴压以一种温和的方式使身体恢复到和谐的状态，有助于解决健康问题。若根据痰湿质的特点制定耳穴贴压针对EH痰湿质患者的调体方案，通过耳穴贴压调体干预以改善EH痰湿质患者的血压和体质，可体现“中国式的个体化治疗”。近年来，耳穴贴压已被广泛证明可以降低EH患者的血压，但其个体化防治EH的微观机制还有待进一步明确。

**3. 耳穴贴压对EH痰湿质患者调体降压的作用机制可能与RAS双轴有关，其机制可能涉及ACE、AngⅡ、AT1R、ACE2、Ang (1-7)、MasR等途径**

肾素-血管紧张素系统（Renin-Angiotensin System, RAS）是EH的发病机制之一。其中经典的体液调节轴为ACE-AngII-AT1R轴，由血管紧张素转换酶（ACE），血管紧张素（AngⅠ、AngⅡ、Ang Ⅲ）及其受体（AT1R、AT2R等）组成。近年来发现的RAS系统新成员，血管紧张素转换酶2（ACE2）、血管紧张素(1-7)[Ang (1-7)]与其受体（MasR）三者构成了RAS新轴，即ACE2- Ang (1-7)- MasR轴。

在经典的RAS轴中，首先由肾脏分泌Renin，切割AGT，获得Ang I，然后通过升高ACE活性，介导AngⅠ形成AngⅡ，AngⅡ作用于两种受体，即AT1R和AT2R。AngⅡ通过AT1R促使血管收缩、水钠潴留、血管平滑肌细胞增生等，从而升高血压。与AT1R不同，AngⅡ通过AT2R发挥强大的抗高血压作用，被激活的AT2R可通过抑制肾素的生物合成和球旁细胞分泌, 引起血管舒张和尿钠排泄, 以降低血压。

而在RAS的新轴中，ACE2切割Ang II生成Ang (1-7)，或与ACE竞争催化共同底物Ang I，生成Ang (1-9)，Ang (1-9)再通过ACE的作用生成Ang (1-7)，Ang (1-7)的主要受体为Mas。Ang (1-7)可通过以下3种途径发挥降压的功效：（1）通过抑制ACE减少AngⅡ的生成，从而减弱AngⅡ的作用；（2）通过释放缓激肽、一氧化氮、前列腺素以及内皮超极化因子等促血管舒张因子，发挥扩张血管、抑制心血管重构以及抗氧化应激等作用；（3）通过增加前列环素I2的释放、环腺苷酸的生成，抑制血管平滑肌细胞的增殖，增加一氧化氮的释放达到拮抗AngⅡ的目的，完成血压的调节。

既往研究证实针灸可通过调节ACE、AT1R和AT2R来抑制自发性高血压大鼠高血压的发展，还可通过下调ACE-AngII-AT1R通路，上调ACE2-Ang (1-7)-MasR通路来调节自发性高血压大鼠的焦虑样行为和记忆障碍。加之已有研究表明耳穴贴压可能可通过上调ACE和下调Ang (1-7)蛋白含量来改善EH阴虚质患者的血压，因此，有理由认为RAS在耳穴贴压调体降压中发挥着重要作用，进一步推测耳穴贴压可能是通过调节RAS双轴上的ACE、AngⅡ、ACE2、Ang (1-7)的蛋白含量对EH痰湿质患者发挥调体降压的作用。

**4. 不同基因型的痰湿质高血压患者对耳穴贴压的敏感性可能不同**

已有综述通过归纳总结现有临床研究文献，发现耳穴贴压可较好地降低血压并改善相关症状，但同时也有较多研究中存在一些患者对耳穴贴压不敏感的情况。此外，多项研究显示抗EH药物疗效与RAS双轴关键基因多态性有关，黄红等的研究表明，在ACE基因不同基因型的EH患者中，厄贝沙坦降压疗效、治疗前后血浆RAS活性降低程度具有差异性，表现为DD型>ID型>II型，由此可知ACE I/D多态性可能是影响EH患者对厄贝沙坦降压反应的重要指标。龚洪涛等通过应用聚合酶链式反应（PCR）对EH患者AT1R基因1166A/C、-810A/T和-521C/T多态性进行分析，结果显示AT1R基因-521C/T CC基因型、CT+TT基因型应用替米沙坦前后舒张压降幅比较的差异有统计学意义（P<0.05），表明AT1R基因-521C/T多态性能独立预测EH患者对替米沙坦反应的个体差异。门琛等探讨EH患者AT2R A1675G基因多态性对氯沙坦降压疗效的影响，发现A等位基因可能是影响该药物降压效果的关键因素。综上可知，基因多态性决定了抗EH药物作用的差异, 从而产生药物治疗效果的个体差异，由此推测基因多态性也有可能影响耳穴贴压的抗血压疗效，但目前尚无相关研究报道不同基因型EH患者与耳穴贴压降压效果的关系。本研究拟检测痰湿质EH患者RAS关键基因的多态性，分析不同基因型、等位基因对耳穴贴压的敏感性，为提高耳穴贴压抗EH疗效提供参考。

**六、研究目的**

1. 对比两组耳穴贴压干预前后RAS双轴蛋白含量变化，揭示耳穴贴压调体降压的分子机制。

2. 分析干预组不同基因型耳穴贴压干预前后的血压的变化，以确定基因型是否影响耳穴贴压治疗降压后的血压降低反应。进一步探讨对耳穴贴压敏感的RAS基因。

**七、纳入和排除标准**

**纳入标准：**

- 年龄45~74岁，性别不限；
- 中医体质为痰湿质；
- 根据国际高血压病学会(ISH)制定高血压病诊断和分类标准（2018）确诊为EH。

**排除标准：**

- 明显兼夹两种及以上体质类型；
- 对耳穴贴压过敏者；
- 耳廓有炎症者；
- 服用含血管紧张素转换酶抑制剂、血管紧张素受体拮抗剂类抗高血压药物者；
- 既往不规律服药，服药依从性差；
- 高血压伴有心、脑、肾等严重并发症的患者；
- 妊娠期和哺乳期妇女；
- 有精神疾病史者；
- 恶性肿瘤患者；
- 参加其他EH药物临床试验。

**八、设计方案**

随机对照试验，平行设计


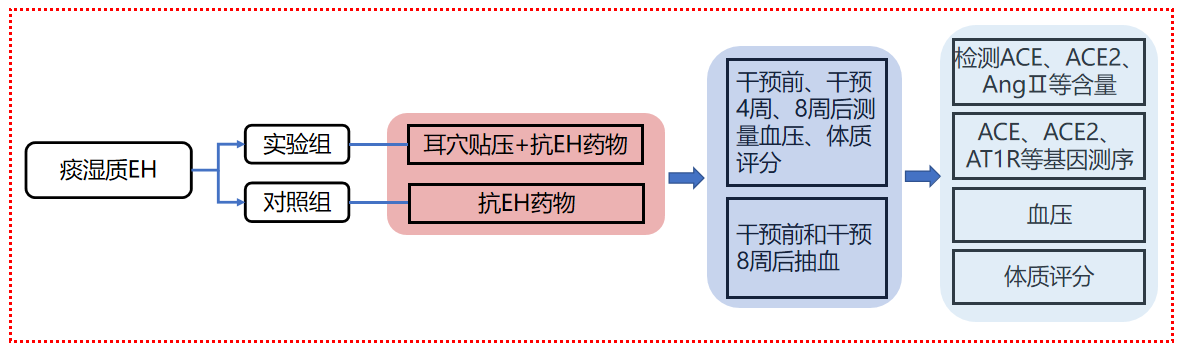


**九、样本量估算**

根据课题组先前的研究中耳穴贴压对EH阴虚质患者血压的疗效，计算本次研究中的样本量。其中干预组干预8周后显效率为28%，对照组为0%，取α=0.05，β=0.10，则：*p*_1_=0.28, *p*_2_=0,‾*p*=(*p*_1_+*p*_2_)/2，代入公式：

$$n=\frac{{2\bar{p}\bar{q}\left( z_{\alpha}+z_{\beta} \right)}^{2}}{\left( p_{1}-p_{2} \right)^{2}}$$

计算出每组至少需要33例，考虑到20%的失访率，每组需40例，共80例。

**十、随机和隐蔽分组的方法**

由不参加纳入病例和后续干预、评估的专人运用SPSS统计软件产生随机序列和分配序列，将分配序列放入不透光的信封中。符合标准的患者按入组顺序领取信封，交至主治医师。随机序列和分配序列的执行人不参与临床患者的纳入、治疗及评价，以确保随机隐藏的充分执行。

**十一、盲法**

参与者将被单独治疗，结局指标评估者和统计人员对小组分配不知情。

**十二、测量指标**

**主要指标：**

**耳穴贴压的效果**：干预前、干预后4周、8周后测量血压。参照中华人民共和国卫生部制定的《中药新药治疗高血压病的临床疗效评定》的判断标准。显效：舒张压下降≥10mmHg并降至正常或下降20mmHg以上；有效：舒张压下降虽未达到10mmHg，但降至正常或下降 10～20mmHg；无效：未达到有效水平。血压测量要求如下：

①测量时间均为下午，保持室内安静，温度为22℃～24℃，且所有血压监测均由受过训练的同一人完成。

②选择经国际标准方案认证合格和袖带标准的上臂式医用电子血压计，并在测量前确保已校准。

③测血压前，受试者应至少坐位安静休息5分钟，30分钟内禁止吸烟，禁饮咖啡和茶水，并排空膀胱。

④受试者坐靠背椅，取坐位，双脚平放于地板，上臂放在桌面上，上臂中点与心脏处在同一水平。

⑤将袖带缚在被测者的上臂，以可插入1～2指为宜，袖带的下缘应在肘窝上2.5cm。

⑥应间隔1-2分钟重复测量，取两次读数的平均值记录。如果收缩压或舒张压的2次读数相差5 mmHg以上，则再次测量，取3次读数的平均值记录。

⑦读取血压数值时，末位数值只为0、2、4、6、8，不可出现1、3、5、7、9。

⑧首诊测量双上臂血压，以后通常测量读数较高的一侧。每次测量2次，应间隔1-2分钟，取两次读数的平均值记录。如果收缩压或舒张压的2次读数相差10mmHg以上，则再次测量，取后两次读数的平均值记录。

**次要指标：**

（1）**痰湿质评分**：干预前、干预后4周、8周后测量痰湿质评分。由中医师采用《中医体质分类与判断》量表，评价干预前、干预后4周、8周后痰湿质分数的变化。

（2）**RAS双轴关键基因表达产物ACE、AngⅡ、ACE2、Ang (1-7)**：干预前、干预后8周测定。对符合纳入标准的EH患者，从肘静脉采集外周静脉血，嘱采集样品前1天禁酒和劳累，女性避开月经期，采用离心机分离血清和血细胞，取血清于-80℃冰箱保存，血清作ELISA检测包括ACE、AngⅡ、ACE2、Ang (1-7)。

（3）**不同基因型的办公室血压：**只有干预组的参与者才会在基线时进行基因多态性测试。对5个单核苷酸多态性（ACE、ACE2、AT1R、AT2R、Mas）进行基因分型。分析不同基因型的办公室血压的变化，以确定基因型是否影响耳针治疗降压后的血压降低反应。

**十三、不良事件**

耳穴贴压通常被认为是一种安全的干预方法。但参与者的任何不适症状（如头晕、耳廓过敏、溃烂等）。在试验期间，所有不良事件的详细信息都会记录在病例记录表中，研究人员会在每次评估时询问患者并完成不良事件报告。伦理委员会将评估不良事件与干预之间的任何相关性，并就是否应继续进行研究做出决定。

**十四、参试者的征募**

我们将张贴公告栏广告，并联系社区医生来招募参与者。招聘信息将主要包括资格标准和联系方式。一名训练有素的调查人员将负责招募参与者。招聘工作将于2022年9月至2023年2月在上城区清波街道社区卫生服务中心进行。在充分解释了研究程序后，符合资格标准的参与者将提供书面的知情同意。

**十五、参试者一般信息的收集**

实足年龄、身高、腰围、臀围、性别、职业、体重、体质指数BMI、坐位血压（SBP/DBP）、既往病史、家族史、用药史（目前患者高血压用药及其他疾病用药情况）

**十六、 基线指标和观测项目**

实足年龄、身高、腰围、臀围、体重、体质指数BMI、坐位血压（SBP/DBP）、吸烟史和饮酒史

**十七、标准操作规程**

嘱病人取坐位，将耳廓常规消毒后，用XS100-A耳穴探测仪探测耳穴并标记，准确取穴后，有资格且训练有素的耳穴治疗师将耳穴压豆片规格为1cm*1cm的王不留行籽贴敷于上述穴位敏感点上，并用拇指和食指相对按压耳穴，以患者感觉局部酸胀发热，皮肤微红为准。每个穴位按压20~30次，使患者感胀痛及耳廓发热。每日早中晚3次。每次单耳贴压，两耳轮流，每3日更换1次耳穴贴压片，连续8周。

**十八、统计分析方法**

使用SPSS 25.0软件对数据进行分析，*P*<0.05被认为具有统计学意义。连续变量将用均值和标准差表示，或用中位数和四分位数表示。分类变量将用频率表示。连续变量采用*t*检验或Wilcoxon秩和检验进行分析。各组治疗前后的差异将采用配对*t*检验来确定。采用卡方检验或Fisher精确检验来比较干预组和对照组之间的有效率。

**十九、参试者管理制度**

- 记录受试者的姓名、联系方式等信息，对信息保密。
- 添加受试者的微信，干预组拉入群聊，每日定时提醒按压耳穴。
- 试验开始前，研究者充分告知受试者试验情况，尊重受试者意愿并签署知情同意书。

**二十、标本管理**

- 每位受试者的血样都做好标签，标注清楚并保存于4℃的冰盒中。
- 运输至实验室后存于-80℃冰箱备用。

**二十一、数据管理制度**

在数据收集过程中，将对所有研究人员进行统一培训，以确保数据质量。参与者的数据将被记录在病例报告表（CRF）中。如果研究人员需要改变CRF的数据，他们需要向主要研究者报告。任何修改都应注明明确的理由，并需要签字并注明日期。两名训练有素的研究人员将把数据输入到一个Excel中，并进行两次输入和距离检查。

**二十二、 原始数据共享计划**

毕业论文发表后可联系导师进行获取

**二十三、试验结束后对参试者的治疗和管理**

对未接受耳穴贴压的对照组通过免费的2个月耳穴贴压服务
